# Van Wyk-Grumbach Syndrome: A Rare Triad of Precocious Puberty, Pituitary Pseudomacroadenoma, and Multicystic Ovaries in an Eight-Year-Old Girl

**DOI:** 10.7759/cureus.116300

**Published:** 2026-09-15

**Authors:** Prashant Bhosale, Rubanbalaji G., Yogendra N Maurya, Anita Meena, Ripdaman Kaur

**Affiliations:** 1 Pediatrics, Deen Dayal Upadhyay Hospital, New Delhi, IND

**Keywords:** idiopathic precocious puberty, pituitary hyperplasia, primary hypothyroidism, severe short stature, van wyk-grumbach syndrome

## Abstract

Van Wyk-Grumbach syndrome (VWGS) is a rare manifestation of chronic, severe primary hypothyroidism in children. It is classically characterized by delayed bone age, short stature, and isosexual precocious puberty, often accompanied by multicystic ovaries and pituitary hyperplasia. Because of its presentation, it is frequently misdiagnosed as an ovarian or pituitary neoplasm, leading to unnecessary surgical interventions. We report the case of an eight-year-old female child who presented with a three-day history of per vaginal bleeding. Physical examination revealed severe disproportionate short stature. Imaging initially suggested neoplastic etiologies, revealing bilateral complex cystic ovarian lesions on abdominal magnetic resonance imaging (MRI) and a sellar/suprasellar mass on brain MRI suggestive of a pituitary macroadenoma. However, a severely delayed bone age (3.5-4 years) and a thyroid profile confirming autoimmune primary hypothyroidism led to the diagnosis of VWGS. The patient was managed purely medically with cautious levothyroxine replacement therapy. This case highlights the critical importance of recognizing VWGS to avoid devastating and unnecessary ovarian or pituitary surgeries.

## Introduction

First described in 1960 by Judson Van Wyk and Melvin Grumbach [1], Van Wyk-Grumbach syndrome (VWGS) is an uncommon but highly specific pediatric endocrine disorder. It arises because of prolonged, untreated, and severe primary acquired hypothyroidism. The clinical hallmark of VWGS is paradoxical: while children with hypothyroidism typically present with delayed puberty [2], patients with VWGS present with isosexual incomplete gonadotropin-independent precocious puberty alongside profoundly delayed bone age and short stature [3].

Other key features include the development of large, bilateral multicystic ovaries and significant pituitary hyperplasia. These findings may mimic ovarian neoplasms and pituitary neoplasms on imaging, potentially leading to unnecessary surgical intervention. Importantly, ovarian enlargement and pituitary hyperplasia are reversible with thyroid hormone replacement. Herein, we present the case of an eight-year-old girl with VWGS whose presentation illustrates the pathophysiological cascades of severe hypothyroidism and underscores the necessity of non-surgical management.

## Case presentation

History and examination

An eight-year-old female child was brought to the outpatient department by her parents with a chief complaint of per-vaginal bleeding lasting three days. The parents reported no other associated complaints. There was no significant past medical history, and the birth history was uneventful. The child had achieved age-appropriate developmental milestones.

On physical examination, she showed severe short stature with a height of 96 cm (<3rd percentile; Z-score < -4.5 SD), a weight of 18 kg (Z-score -2.24 SD), and an immature upper segment to lower segment (US:LS) ratio of 1.2:1  (reference ratio for age: ~1.0:1), reflecting delayed skeletal maturation and infantile body proportions rather than an intrinsic skeletal dysplasia. Her calculated body mass index (BMI) was 19.5 kg/m² (95th percentile). Her mid-parental height was calculated as 151.5 cm (maternal height: 148 cm; paternal height: 168 cm) with a target height range of 143-160 cm, confirming pathologic rather than familial short stature. Secondary sexual characteristic examination revealed no breast enlargement (Sexual Maturity Rating (SMR) 1) and no pubic hair development (SMR 1) despite the presence of vaginal bleeding, consistent with incomplete peripheral precocious puberty (Figure 1).

**Figure 1:**
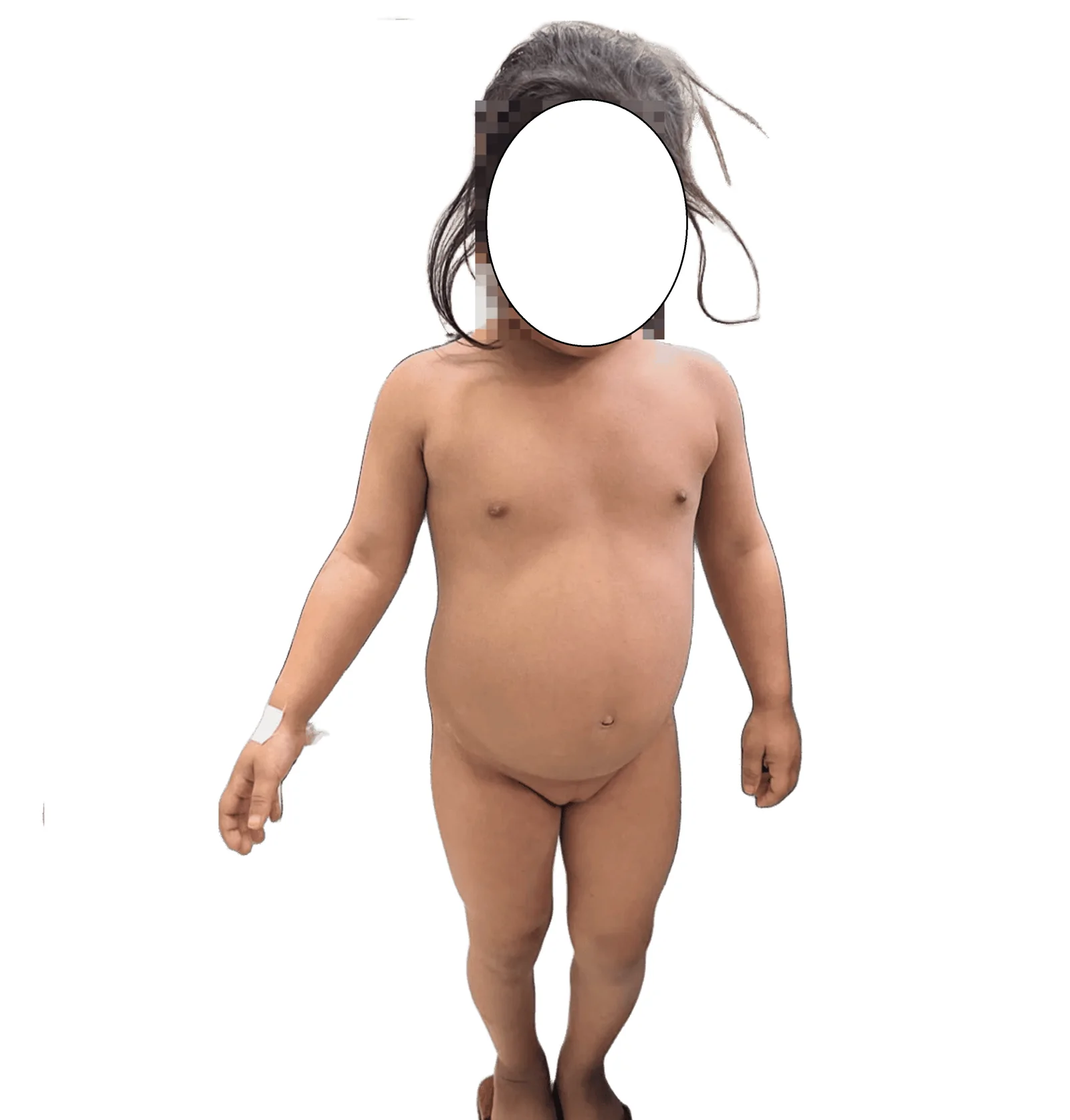
Clinical photograph of the patient demonstrating disproportionate short stature and Sexual Maturity Rating (SMR) 1 (absence of breast development and pubic hair) Photograph used with written permission from parents

A detailed perineal and local genital examination revealed no evidence of vulvovaginal trauma, foreign body, laceration, excoriation, or local neoplastic lesions. Abdominal examination was soft and non-tender, with no signs of acute peritoneal irritation, guarding, or rigidity to suggest ovarian torsion.

Diagnostic investigations

Initial workup for vaginal bleeding included routine laboratory investigations, which were unremarkable except for macrocytic anemia (Table 1). Severe hypothyroidism can be associated with anemia and, in some patients, macrocytosis because of impaired erythropoiesis and altered red-cell maturation. In this patient, the absence of documented vitamin B12 or folate deficiency, together with severe untreated hypothyroidism, suggested that the macrocytic anemia was associated with the underlying hypothyroid state.

**Table 1:** Initial laboratory investigations

Parameter	Patient Value	Reference Range
Hemoglobin	8.3 g/dL	11.5–13.5 g/dL
Leukocytes	8080 cells/cu.mm	6000–15000 cells/cu.mm
Mean corpuscular volume	107.4 fL	80–100 fL
Urine red blood cells	Nil	Nil
Serum urea	29 mg/dL	15–45 mg/dL
Serum creatinine	0.5 mg/dL	<1.2 mg/dL
Serum sodium	140 mmol/L	135–150 mmol/L
Serum potassium	4.5 mmol/L	3.5–5 mmol/L
Serum chloride	106 mmol/L	90–110 mmol/L

Evaluation for short stature included bone age assessment via a left wrist radiograph, which demonstrated a delayed bone age of 3.5-4 years against a chronological age of eight years. Subsequent endocrine and biochemical workup confirmed autoimmune primary acquired hypothyroidism (Table 2).

**Table 2:** Endocrine and biochemical evaluation for short stature

Parameter	Patient Value	Reference Range
Total serum calcium	9 mg/dL	9–11 mg/dL
Serum phosphate	4.8 mg/dL	4–5.6 mg/dL
Alkaline phosphatase	113 IU/L	108–306 IU/L
Serum folate	9.8 ng/mL	3.5–20 ng/mL
Serum vitamin B12	284 pg/mL	180–914 pg/mL
Free triiodothyronine	0.56 pg/mL	2.3–4.2 pg/mL
Free thyroxine	0.21 ng/dL	0.8–1.8 ng/dL
Thyroid-stimulating hormone	>1500 mIU/L	0.5–5 mIU/L
Anti-thyroid peroxidase antibody	310.9 IU/mL	<35 IU/mL
Anti-thyroglobulin antibody	125.5 IU/mL	20–40 IU/mL

Due to ongoing vaginal bleeding, transabdominal ultrasonography (USG) was performed, revealing complex cystic lesions in the right and left ovaries measuring 139 mL (8.1 cm × 4.9 cm × 6.7 cm) and 15 mL (3.3 cm x 2.3 cm x 1.0 cm), respectively, with an endometrial thickness of 4.5 mm. Abdominal magnetic resonance imaging (MRI) showed well-defined, thin-walled cystic lesions with internal septations in the right adnexa/iliac fossa and left adnexa (Figure 2). Color Doppler evaluation demonstrated preserved ovarian stromal vascularity with normal arterial and venous waveforms, effectively ruling out adnexal torsion despite the marked cystic enlargement.

**Figure 2:**
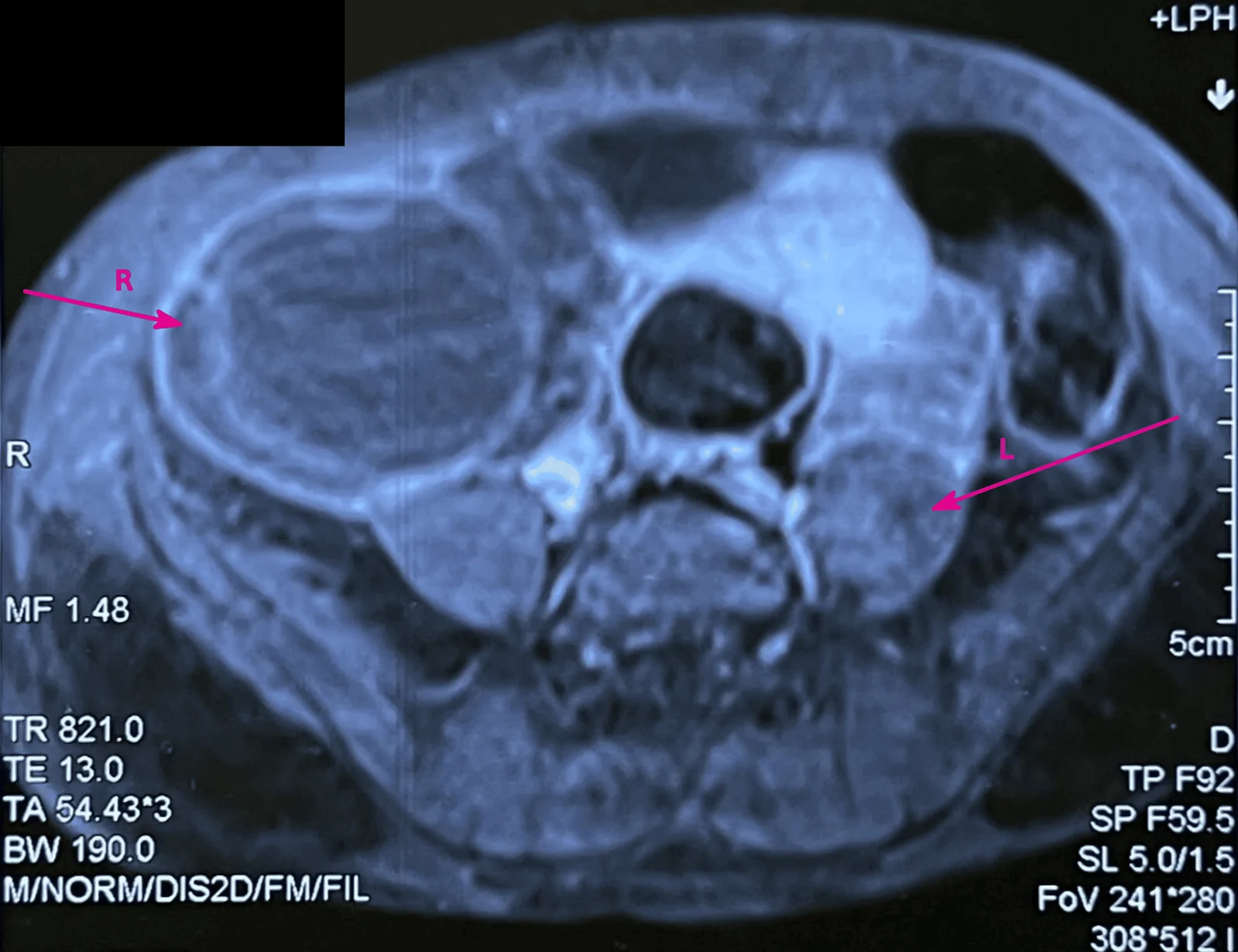
MRI Abdomen showing cystic lesions with internal septations in right (arrow R) and left adnexa (arrow L). Axial T2-weighted turbo spin-echo with fat saturation, post-contrast image

Given the initial radiological concern for a neoplastic process, hormonal and tumor markers were evaluated (Table 3). Although mild elevations in serum cancer antigen (CA)-125 (49.3 U/mL) and carcinoembryonic antigen (CEA) (6.14 ng/mL) were noted, markers specific for malignant germ cell tumors, including beta-human chorionic gonadotropin (β-hCG) and CA 19-9, remained within normal limits, and alpha-fetoprotein (AFP) showed only a negligible borderline elevation. These elevations were interpreted as non-specific, reactive peritoneal and epithelial changes secondary to massive mechanical distension from bilateral multicystic ovaries rather than true neoplastic transformation.

**Table 3:** Serum hormonal and tumor marker profiles

Parameter	Patient Value	Reference Range
Luteinizing hormone	0.06 mIU/mL	<0.3 mIU/mL
Follicle-stimulating hormone	9.76 mIU/mL	<2.9 mIU/mL
Estradiol (Estrogen)	380.51 pg/mL	10–20 pg/mL
Cancer antigen 125 (CA 125)	49.3 U/mL	<35 U/mL
Carcinoembryonic antigen (CEA)	6.14 ng/mL	<3 ng/mL
Cancer antigen 19-9 (CA 19-9)	8.4 U/mL	<37 U/mL
Alpha-fetoprotein	13.71 ng/mL	<10 ng/mL
Beta-human chorionic gonadotropin (β-hCG)	1.84 mIU/mL	<5 mIU/mL

Brain MRI revealed a sellar and suprasellar soft tissue lesion measuring 21 mm x 11 mm x 17.8 mm, displaying a characteristic figure-of-eight appearance with superior displacement of the optic chiasm (Figure 3). The lesion exhibited homogeneous post-contrast enhancement and abutted the bilateral internal carotid arteries with preserved vascular flow. Although initially interpreted radiologically as a pituitary macroadenoma, the absence of visual field defects and the presence of severe primary hypothyroidism favored pituitary thyrotrope hyperplasia. A modestly elevated serum prolactin level of 111 ng/mL (reference range: 3.0-20.0 ng/mL; more than 200 ng/mL is suggestive of neoplasia) supported this diagnosis.

**Figure 3:**
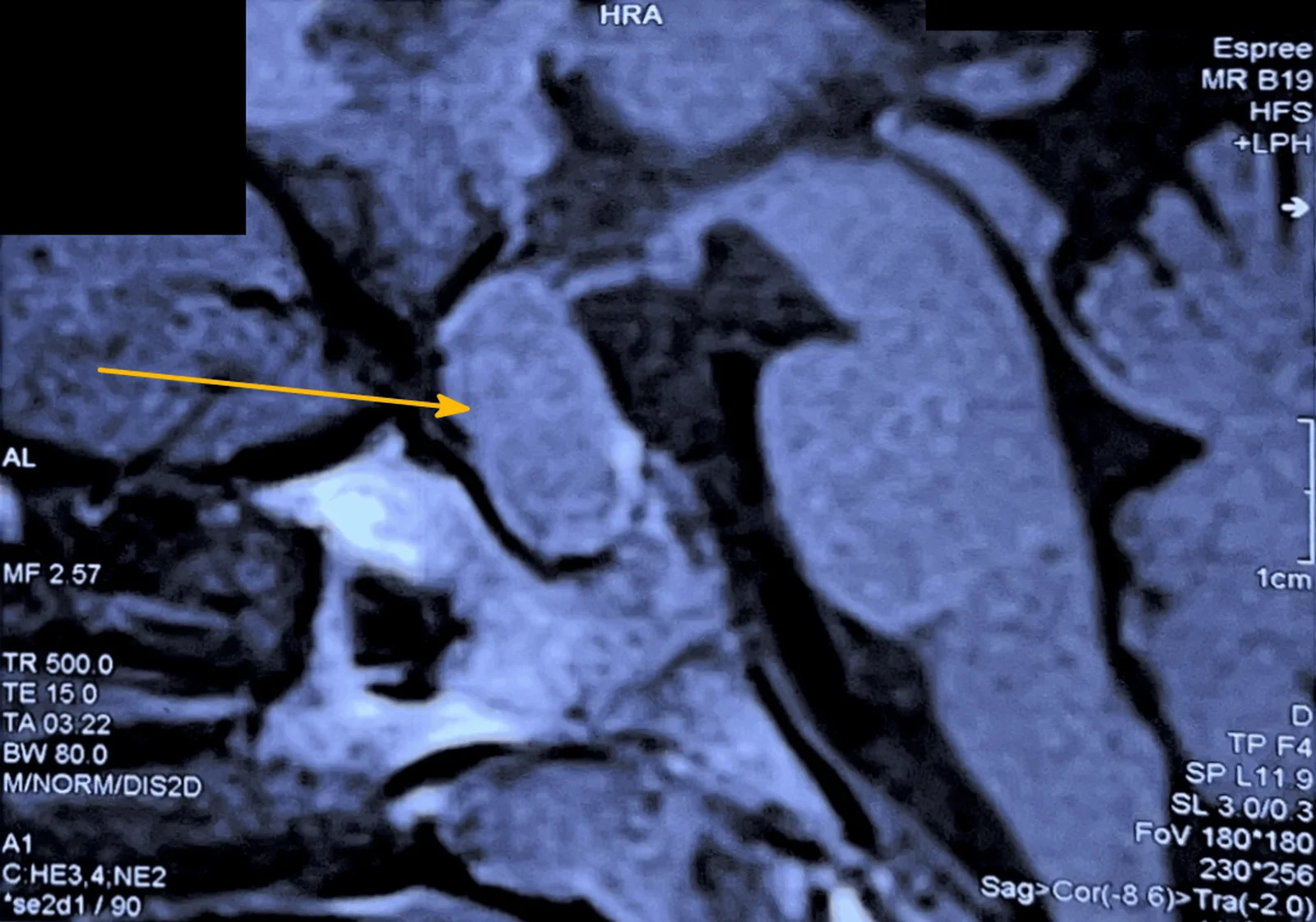
MRI Brain showing "figure-of-eight" appearance with superior displacement of the optic chiasm. Mid-sagittal T1-weighted spin-echo contrast-enhanced MRI

Integrating the clinical presentation of severe short stature, delayed bone age, isosexual precocious puberty, multicystic ovaries, and pituitary enlargement in the context of severe autoimmune hypothyroidism established the diagnosis of VWGS.

Treatment and follow-up

The patient was managed medically with oral levothyroxine at an initial conservative dose of 50 mcg once daily (~3 µg/kg/day), administered on an empty stomach. Cautious dosing was maintained to avoid precipitating cardiac compromise or pseudotumor cerebri.

Per vaginal bleeding ceased on day three, temporally aligning with the start of oral levothyroxine therapy. The patient was discharged on day 10 in a stable condition and scheduled for multidisciplinary outpatient follow-up. Repeat pelvic ultrasonography showed substantial reduction in endometrial thickness and ovarian volumes (right ovary: 6.9 mL, 3.3 cm × 2.0 cm x 1.9 cm; left ovary: 2.1 mL, 2.0 cm × 1.1 cm x 1.8 cm). Follow-up thyroid function testing demonstrated marked biochemical improvement (Table 4).

**Table 4:** Follow-up thyroid profile after one month of levothyroxine replacement therapy.

Parameter	Patient Value	Reference Range
Free triiodothyronine (FT3)	4.01 pg/mL	2.3–4.2 pg/mL
Free thyroxine (FT4)	1.24 ng/dL	0.8–1.8 ng/dL
Thyroid-stimulating hormone (TSH)	6.67 mIU/L	0.5–5 mIU/L

Repeat brain MRI performed two months after starting therapy revealed marked regression of the previously identified sellar and suprasellar enlargement (Figure 4). The patient also exhibited significant catch-up growth, reaching a height of 101 cm at six months of treatment.

**Figure 4:**
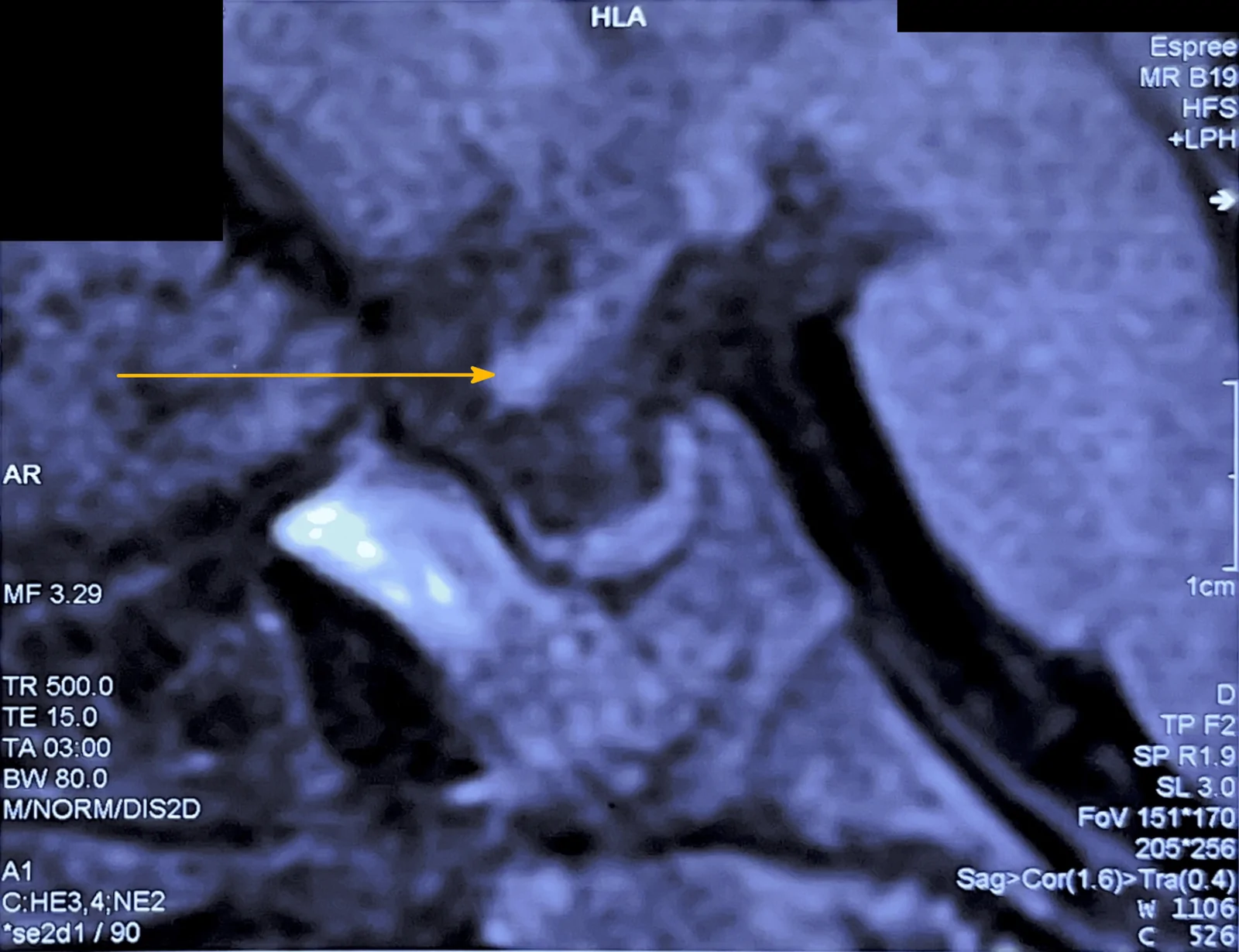
MRI brain after levothyroxine therapy showing resolution of pituitary enlargement. Mid-sagittal T1-weighted spin-echo contrast-enhanced MRI

## Discussion

VWGS is a classic example of overlapping endocrine feedback loops. The pathophysiology of this syndrome is driven by the loss of negative feedback caused by chronic, severe primary hypothyroidism (low triiodothyronine (T3) and thyroxine (T4) levels).

The deficiency in circulating thyroid hormones induces a marked compensatory overproduction of thyrotropin-releasing hormone (TRH) by the hypothalamus, driving excessive secretion of thyroid-stimulating hormone (TSH) from the anterior pituitary. Because TSH, follicle-stimulating hormone (FSH), luteinizing hormone (LH), and human chorionic gonadotropin (hCG) share a common alpha-subunit [4], markedly elevated serum TSH levels exhibit cross-reactivity and weak agonistic activity at ovarian FSH receptors. This receptor cross-activation promotes follicular development, formation of multicystic ovaries, and hypersecretion of estradiol. The resulting estrogen surge triggers isosexual precocious puberty, presenting with thelarche and withdrawal vaginal bleeding (menarche).

Concurrently, elevated hypothalamic TRH stimulates lactotroph cells, resulting in hyperprolactinemia [5]. Elevated prolactin levels suppress gonadotropin-releasing hormone (GnRH) pulsatility and inhibit LH secretion, thereby preventing true central pubertal progression and confining the clinical presentation to isolated estrogen-dependent effects.

Prolonged hyperstimulation by TRH also leads to hypertrophy and hyperplasia of anterior pituitary thyrotropes and lactotrophs. Radiographically, pituitary hyperplasia typically appears as a smooth, symmetrically enlarged sellar/suprasellar mass demonstrating homogeneous post-contrast enhancement-features that are frequently mischaracterized as a non-functioning pituitary macroadenoma [6], as occurred initially in this case.

Differentiating physiologic pituitary hyperplasia from a neoplastic macroadenoma is essential to prevent inappropriate surgical or radiotherapeutic intervention [7]. While macroadenomas represent true clonal neoplasms requiring specialized interventions, thyrotrope hyperplasia is entirely reversible with thyroid hormone replacement therapy. Biochemically, hyperplasia is characterized by markedly elevated TSH levels alongside mild-to-moderate hyperprolactinemia, whereas non-TSH-secreting macroadenomas typically present with normal or suppressed TSH values. On MRI, hyperplasia typically demonstrates symmetric, homogeneous enhancement with preserved sellar contours, in contrast to the irregular, heterogeneous architecture frequently seen in true adenomas.

In prepubertal girls presenting with vaginal bleeding and large pelvic masses, ruling out critical surgical emergencies, particularly ovarian torsion, local trauma, and functional ovarian neoplasm, is of paramount importance. The preservation of robust internal vascular perfusion on color Doppler and the lack of acute peritoneal signs help exclude torsion. Furthermore, while the presence of multi-locular cystic masses and elevated estradiol frequently triggers concern for granulosa cell or malignant germ cell tumors, clinicians should be aware that mild elevations in CA-125 and CEA can represent reactive, non-malignant mesothelial and epithelial distension from large benign cysts. Misinterpreting multi-cystic ovaries or sellar masses as neoplasms poses a risk of unwarranted oophorectomy or transsphenoidal hypophysectomy.

The dramatic radiological and clinical regression following levothyroxine replacement alone provides retrospective confirmation of the non-neoplastic, hypothyroid etiology, reaffirming that aggressive surgical resection or biopsy should be deferred when clinical and endocrine hallmarks of VWGS are present. Following the initiation of oral levothyroxine replacement, clinical and radiographic recovery follows a predictable timeline. Vaginal bleeding typically ceases within days to weeks, secondary sexual characteristics gradually regress, ovarian cysts undergo complete resolution within two to six months, serum TSH normalizes, and sellar enlargement regresses over weeks to months, ultimately restoring normal pituitary morphology [8,9].

## Conclusions

VWGS represents a critical diagnostic challenge in which chronic, severe primary hypothyroidism masquerades as precocious puberty alongside suspected pituitary and ovarian tumors. A high index of suspicion-triggered by the hallmark combination of profound short stature, drastically delayed bone age, and sexual precocity-is essential to reach the correct diagnosis. Recognizing this rare syndrome prevents unnecessary and irreversible surgical interventions, such as oophorectomy or transsphenoidal hypophysectomy. In this patient, conservative medical therapy with oral levothyroxine was associated with prompt cessation of vaginal bleeding, marked regression of multicystic ovarian enlargement, improvement in thyroid parameters, and radiological resolution of pituitary hyperplasia during short-term follow-up. While these findings highlight the dramatic responsiveness of this condition to thyroid hormone replacement alone, continued long-term endocrine follow-up remains necessary to monitor sustained pubertal progression, complete ovarian cyst resolution, and final adult height outcomes.
